# Bringing light onto the Raunkiæran shortfall: A comprehensive review of traits used in functional animal ecology

**DOI:** 10.1002/ece3.10016

**Published:** 2023-04-19

**Authors:** Thiago Gonçalves‐Souza, Leonardo S. Chaves, Gabriel X. Boldorini, Natália Ferreira, Reginaldo A. F. Gusmão, Phamela Bernardes Perônico, Nathan J. Sanders, Fabrício B. Teresa

**Affiliations:** ^1^ Department of Biology, Ecological Synthesis and Biodiversity Conservation Lab Federal Rural University of Pernambuco Recife Brazil; ^2^ Department of Ecology and Evolutionary Biology University of Michigan Ann Arbor Michigan USA; ^3^ School for Environment and Sustainability, Institute for Global Change Biology University of Michigan Ann Arbor Michigan USA; ^4^ Graduate Program in Ethnobiology and Nature Conservation, Department of Biology Federal Rural University of Pernambuco Recife Brazil; ^5^ Escola de Educação e Humanidades Universidade Católica de Pernambuco Recife Brazil; ^6^ Graduate Program in Biodiversity, Department of Biology Federal Rural University of Pernambuco Recife Brazil; ^7^ Graduate Program in Natural Resources of Cerrado State University of Goiás Anápolis Brazil; ^8^ Biogeography and Aquatic Ecology Lab State University of Goiás Anápolis Brazil

**Keywords:** functional traits, knowledge shortfalls, Raunkiæran shortfall, response and effect traits, trait‐based ecology

## Abstract

Trait‐based approaches elucidate the mechanisms underlying biodiversity response to, or effects on, the environment. Nevertheless, the Raunkiæran shortfall—the dearth of knowledge on species traits and their functionality—presents a challenge in the application of these approaches. We conducted a systematic review to investigate the trends and gaps in trait‐based animal ecology in terms of taxonomic resolution, trait selection, ecosystem type, and geographical region. In addition, we suggest a set of crucial steps to guide trait selection and aid future research to conduct within and cross‐taxon comparisons. We identified 1655 articles using virtually all animal groups published from 1999 to 2020. Studies were concentrated in vertebrates, terrestrial habitats, the Palearctic realm, and mostly investigated trophic and habitat dimensions. Additionally, they focused on response traits (79.4%) and largely ignored intraspecific variation (94.6%). Almost 36% of the data sets did not provide the rationale behind the selection of morphological traits. The main limitations of trait‐based animal ecology were the use of trait averages and a rare inclusion of intraspecific variability. Nearly one‐fifth of the studies based only on response traits conclude that trait diversity impacts ecosystem processes or services without justifying the connection between them or measuring them. We propose a guide for standardizing trait collection that includes the following: (i) determining the type of trait and the mechanism linking the trait to the environment, ecosystem, or the correlation between the environment, trait, and ecosystem, (ii) using a “periodic table of niches” to select the appropriate niche dimension to support a mechanistic trait selection, and (iii) selecting the relevant traits for each retained niche dimension. By addressing these gaps, trait‐based animal ecology can become more predictive. This implies that future research will likely focus on collaborating to understand how environmental changes impact animals and their capacity to provide ecosystem services and goods.

## Graphical abstract

Most studies investigated in this review concentrated in vertebrates, terrestrial habitats, the Palearctic realm. Almost 36% of the datasets did not provide the rationale behind the selection of morphological traits. The main limitations of trait‐based animal ecology were the use of trait averages and a rare inclusion of intraspecific variability. Nearly one‐fifth of the studies based only on response traits conclude that trait diversity impacts ecosystem processes or services without justifying the connection between them or measuring them.

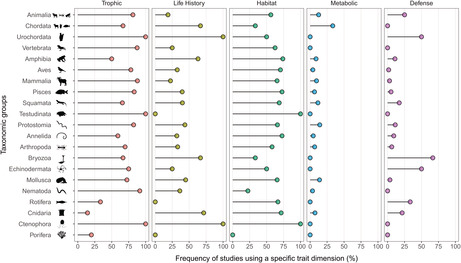

## 1 INTRODUCTION

“Vive la différence” is an expression used when someone wants to celebrate a difference between people or things. Díaz and Cabido (2001) employed this expression in their seminal study, producing the first synthesis of how plant trait variation affects ecosystem functioning (the so‐called *effect traits*: Lavorel & Garnier, 2002). Likewise, trait differences are also relevant to measure how species respond to environmental changes (i.e., *response traits*: Lavorel & Garnier, 2002). Despite the fact that these functional trait types were defined in the late 1990s and early 2000s (Díaz & Cabido, 2001; Lavorel & Garnier, 2002; Naeem et al., 1994; Tilman, 1999), the use of functional traits to examine species response to or impact on the environment in studies of plants and animals can be traced back to early ecologists such as Elton, Humboldt, Hutchinson, Grime, Raunkiaer, and Root (Garnier et al., 2016; Malaterre et al., 2019). Even though trait‐based studies with plants and animals have been conducted for over a century, this research program has advanced significantly more for plants than animals (but see recent advances in Schleuning et al., 2023). For example, the first global protocol for standardizing plant traits measurements was published in the early 2000s (Cornelissen et al., 2003), but the first global protocols for standardizing traits for animals were published very recently, for example, Pey et al. (2014) for soil invertebrates, Schmera et al. (2015) for macroinvertebrates, Moretti et al. (2017) for terrestrial invertebrates, and Tobias et al. (2022) for birds. Recent research has criticized the use of functional traits in animal studies, stating that it is often too arbitrary (Kearney et al., 2021). Therefore, there are two pressing needs to progress trait‐based ecology studies: (1) a thorough examination of the literature on animals to reveal shortcomings in trait selection and the representation of different taxa and geographical regions; (2) a structured approach to guide trait collection and selection in the context of trait‐based studies across various taxonomic groups and regions.

The attempts to uncover gaps in trait‐based literature have primarily concentrated on specific groups of animals or themes, as seen in studies by de Bello et al. (2010), Etard et al. (2020), Hevia et al. (2017), Schmera et al. (2015), Villéger et al. (2017), and Mendes et al. (2021). These studies have highlighted gaps and biases at different levels, such as the frequent use of a limited set of commonly known traits, like body size, while other potentially relevant functional traits remain overlooked (Vitule et al., 2017). Second, in some cases there is a lack of trait information for most species in the community. As an alternative, researchers have used data from the literature and online databases. In these cases, they collect traits that are most easily measured (often morphological traits) and are used as proxies for the specific function of interest or correlate with that function (e.g., Zhu et al., 2017). These strategies are valid alternatives to deal with knowledge deficit. However, they bring some issues that may make traits collected elsewhere not compatible with the organism in the studied site, such as spatiotemporal intraspecific variation (Silva et al., 2019; see also Malaterre et al., 2019). Third, collation of interspecific data is the dominant approach in trait‐based ecology, as studies argue that trait variation between species is larger than within species (McGill et al., 2006). However, there is theoretical and empirical support demonstrating that intraspecific trait variability plays an important role in community assembly and ecosystem processes (Albert et al., 2011; Araújo et al., 2011; Bolnick et al., 2011; Siefert et al., 2015; Violle et al., 2012). Fourth, the asymmetrical research efforts result in a bias in the distribution of animal trait data availability and trait‐based studies across taxonomic groups, regions, and ecosystems (Etard et al., 2020; Hevia et al., 2017; Schleuning et al., 2023). Fifth, there are standardized protocols for organizing and validating trait collation which compromise comparative analyses and data sharing (Hortal et al., 2015).

These biases are directly or indirectly related to the knowledge gap on trait variation within and between species and their ecological function, known as the Raunkiæran shortfall (sensu Hortal et al., 2015). So far, the extent of these biases across animal research literature remains to be evaluated. Therefore, our aim in this broad review is to identify the extent of these previously identified biases and stimulate the research effort to overcome them as part of the challenge of filling the gaps of the Raunkiæran shortfall.

The existing protocols for guidance on applying a trait framework with animals were first proposed to vertebrates (Luck et al., 2012). This work organized vertebrate trait‐based framework providing the following advances: (i) a conceptual definition of traits, (ii) a link between response and effect traits, (iii) the levels in which a trait varies within and between species, (iv) the scale of analysis, and lastly (v) guidelines for trait selection. Moretti et al. (2017) investigated the key response and effect traits in terrestrial invertebrates by reviewing the literature. The authors created a handbook of trait selection and measurement protocols that allow for cross‐taxon comparisons of trait variation in terrestrial invertebrates as response to or effect on the environment. Furthermore, they provided relevant trait definitions and grouped key traits into five categories: morphology, feeding, life history, physiology, and behavior. Similar attempts with animals have been done for vertebrates (Wilman et al., 2014), zooplankton (Hébert et al., 2017; Litchman et al., 2013), aquatic invertebrates (Schmera et al., 2015), terrestrial invertebrates (Brousseau et al., 2018; Moretti et al., 2017), soil invertebrates (Pey et al., 2014), ants (Parr et al., 2017), beetles (de Castro‐Arrazola et al., 2023; Fountain‐Jones et al., 2015), and fish (Frimpong & Angermeier, 2009). These protocols, however, use various approaches to grouping, organizing, collecting, and measuring trait data, which may limit our capacity to improve cross‐taxon comparisons when using many animal taxonomic groups (but see Moretti et al., 2017). Yet, there is a significant hurdle to this standardization, since even in well studied animal groups (e.g., vertebrates), it is difficult to identify a broad but relevant protocol (Luck et al., 2012).

Here, we review the trait‐based ecology literature encompassing virtually all animal taxonomic groups studied to date to evaluate its trends and gaps. Our study covers a wide variety of taxonomic groups (Vertebrata, Ecdysozoa, Protostomia, Spiralia, and Echinodermata) and several terrestrial zoogeographical and marine biogeographical realms. This systematic review aims to specifically identify the trends and gaps in trait‐based studies involving animals in regard to (i) the taxonomic groups, ecosystem types, and geographical regions examined and (ii) the methods used for selecting traits across different taxa. We present the most comprehensive review of literature to date on trait‐based animal ecology and examine its challenges, limitations, and knowledge gaps. Finally, we offer solutions to address the main gaps identified, to assist future studies (1) in selecting an appropriate set of traits, (2) relating traits to species performance and function, and (3) making comparisons of functional traits across taxa and scales.

## 2 MATERIALS AND METHODS

### 2.1 Literature search protocol

We performed a systematic review of published articles on trait‐based animal ecology studies using a search protocol through the online search engines *Scopus* and *Web of Science*. We search the literature in titles, abstracts, and keywords using the following terms: “functional diversity” OR “functional trait*” AND “animal” OR “vertebrate*” OR “invertebrate*” OR “ecdysozoa” OR “protostomia” OR “spiralia” OR “echinodermata” OR “macroinvertebrate,” OR “aves,” OR “amphibia,” OR “reptilia,” OR “chiroptera” OR “cnidaria” OR “porifera” OR “ctenophora” OR “crustacea*” OR “insect*” OR “nematoda” OR “tardigrada” OR “onycophora” OR “annelida” OR “brachiopoda” OR “mollusk*” OR “bryozoa” OR “arthropod” OR “coleoptera” OR “heteroptera” OR “homoptera” OR “lepidoptera” OR “hymenoptera” OR “gastropoda” OR “isopoda” OR “acanthocephala” OR “brachipoda” OR “cycliophora” OR “nemertea” OR “platyhelminthes” OR “sipuncula” OR “diptera” OR “trichoptera” OR “diplopoda” OR “chilopoda” OR “collembola” OR “ostracoda” OR “copepod” OR “rotifera” OR “decapoda” OR “amphipoda” OR “arachnid*” OR “actinopterygii” OR “chondrichthyes” OR “sarcopterygii” OR “tetrapoda” OR “squamata” OR “testudinata” OR “crocodylomorpha” OR “thynchocephalia” OR “echinozoa” OR “crinozoa” OR “asterozoa” OR “mammal*” OR “primate*” OR “bat” OR “ungulate*” OR “monkey” OR “rodent*” OR “amphibian” OR “reptile” OR “lizard” OR “snakes” OR “anuran*” OR “bird” OR “avian” OR “fish” OR “microcrustacean” OR “arachnid,” OR “spider” OR “earthworm*” OR “sponge*” OR “coral*” OR “bee” OR “shrimp” OR “crab” OR “snail” OR “zooplankton” OR “wasp” OR “ant” OR “macrofauna”. We used these taxonomic groups based on the hierarchical classification proposed by Ruggiero et al. (2015). Although this is not an exhaustive list of all recognized taxa in this paper, we chose a variety of Infrakingdom, Superphylum, Phylum, Subphylum, Class, and Order. We also included a list of common names that the authors were familiar with or that appeared in Google Search when those categories were used as search terms. The first search in July 2020 returned 7854 manuscripts.

We did not use keywords typically used in 1980's and 1990's such as “functional group*,” “functional guild*,” “guild*,” “trophic group*,” and “ecomorphology” (e.g., Miles et al., 1987; Winemiller, 1989). These terms represent an earlier approach to assessing patterns of resource use versus competition (e.g., the guild concept in Simberloff & Dayan, 1991) that precede the rise of functional ecology (Díaz & Cabido, 2001; Lavorel & Garnier, 2002). In addition, we did not add “trait‐based” or “biological trait” terms in our search because including them introduced a huge amount of unrelated literature that goes beyond the scope of this manuscript. However, studies that used the terms functional group, functional guild, trophic group, biological trait, trait‐based, or ecomorphology but also incorporated the keywords “functional diversity” or “functional trait” were included in the final database (see Table S1).

We evaluated all 7854 manuscripts in three stages: (1) first, we read manuscript titles and abstracts and selected only those studies using at least one measured trait of animal species. At this stage, we excluded the following:

- conference abstracts, theses, reviews, and methodological papers describing analysis and functional diversity metrics;
- articles using functional traits based on surrogates (e.g., species richness as a proxy of functional diversity, without any explicit collation of a species trait);
- studies using traits of plants, fungi, protists or bacteria solely (without including any animal taxonomic group); and
- articles that used the term “functional trait” to assess the variation in molecules and tissues in the context of histology, neurology, enzymology, or genetics without a clear ecological meaning.

In the second stage, we screened the full text of 2154 manuscripts to confirm if it matches the minimum criteria cited above. As a result, we excluded 621 manuscripts that were not previously removed by reading titles and abstracts, and selected 1655 studies for the extraction stage. In the last stage, the selected articles were then fully read for data extraction (Appendix S1: Figure S1). Because some manuscripts used more than one taxonomic group, we classified data from different taxonomic groups in the same study as separate data sets. Consequently, we extracted data from 1790 data sets in the 1655 studies.

To guarantee a standardized extraction protocol among authors of this study, we first selected 20 random manuscripts to be compared among all authors that independently extracted study data in a “training” spreadsheet. Then, we compared the information agreement and fixed (when necessary) potential issues in the information extracted by the authors. After correcting extraction bias and answering all doubts from authors about the extracted data, we randomly split the 1655 papers into seven blocks that were screened and extracted by seven authors. We organized weekly meetings to discuss potential problems in data extraction (e.g., an unexpected type of study that was not previously discussed among authors) or to solve general questions from one or more authors about that extraction. After the end of this stage, the leading author screened all papers to assess extraction quality and to fix potential incorrect information.

All authors followed a standard spreadsheet to extract the following data from text, figures, tables, and Appendices S1 and S2: (i) the taxonomic unit of trait identification (e.g., subspecies, species, genus, family, multiple units), (ii) the lowest taxonomic resolution that grouped all species registered in the study (e.g., several spider families at the order level, i.e., Araneae), (iii) the most inclusive taxonomic group (e.g., Trichoptera, Araneae, Zooplankton), (iv) the location (e.g., country, ocean, island, global) where the study was carried out, (v) the scale of the study (local, regional/continental or global), (vi) ecosystem type (freshwater, marine or terrestrial), (vii) niche dimension (trophic, life history, habitat, defense, metabolic, and other), (viii) whether the study used response and/or effect traits, (ix) whether the study considered intraspecific variation, and (x) a detailed description of selected traits (Table S1). All the information from *i* to *x* was available in the main manuscript text or Appendices S1 and S2 and, therefore, we did not contact any authors requesting additional data.

### 2.2 Taxonomic unit and group

We used the taxonomic unit informed in each study to determine the refinement of traits, that is, whether a specific trait was attributed to specimens, subspecies, species, genus, and so on. When the authors did not provide the information in the methods, we considered the taxonomic names in traits provided in tables or Appendices S1 and S2 to check the taxonomic unit used in that specific study.

The taxonomic groups used in data extraction (lowest taxonomic unit and the most inclusive taxonomic group) were defined based on the higher‐level classification proposed by Ruggiero et al. (2015). The detailed list provided by these authors was used after the end of data extraction to standardize lower (order or class) and higher (phylum or kingdom) level classification of animal taxonomic groups. Studies using multiple animal groups (e.g., butterflies, beetles, and birds) were classified with the most inclusive hierarchical level. However, some studies were too broad, and the classification was only possible at the Animalia level (0.9%, 15 out of 1655 manuscripts) (Table S2), as they extracted trait information using at least one taxon of vertebrates and invertebrates. Furthermore, these different groups are not nested within each other, so a data set labeled “Animalia” or “Vertebrata” did not necessarily include, for example, Pisces in their research. In fact, it was not possible to create nested taxonomic groups because the studies were independent and dealt with very different combinations of taxonomic groups. A taxonomic group from a higher level, such as Animalia or Chordata, does not always imply a coarse taxonomic unit (and trait resolution) because the taxonomic group indicates the most inclusive hierarchical level to aggregate the study taxa. The taxonomic unit, on the other hand, represents the refinement of trait resolution.

### 2.3 Geographical scale and biogeographic realms

We defined the ecosystem type of each study based on 20 zoogeographical (terrestrial and freshwater ecosystems, Holt et al., 2013) and 30 marine biogeographic realms (Costello et al., 2017). The definition of geographical scale is very complex as studies vary enormously in extent (from a population separated by a few metres to the whole world). Therefore, because this study does not intend to explicitly discuss the potential effect of the geographical extent in trait selection and quality, we simply divided the studies into three scales: (i) local scale, which represents studies performed in a unique locality (e.g., a city, a protected area, etc.) with replicates encompassing metres or few kilometers (<10 km), (ii) regional to continental scale, representing studies whose replicates were distributed across different sites in a landscape or a continent, and (iii) global scale, which includes studies that retrieved data from literature or collected animal traits in at least three continents and two hemispheres. Studies performed on fewer than three continents and one hemisphere were included on the regional to continental scale.

### 2.4 Trait categories and data cleaning

A trait, as broadly defined by McGill et al. (2006), is a measurable property of organisms, typically quantified at the individual level and used for comparisons across species. In addition, a “functional trait” is a trait that influences organismal performance determining the organism's response to pressures and drivers of change and its effects on ecosystem functioning (Díaz & Cabido, 2001; McGill et al., 2006). Functional traits are generally categorized into two groups: response traits, which are linked to an organism's response to environmental factors, and effect traits, which have an impact on ecosystem functioning (Lavorel & Garnier, 2002; Violle et al., 2007). We searched on each study the description of the selected traits to decide whether it used a response and/or effect trait using the following rule: (i) the author explicitly cited that the selected trait is response or effect (or both) and (ii) the authors did not explicitly inform trait type but described in the hypothesis/questions/predictions a clear response or effect relationship.

For instance, we categorized as “response traits” those traits used in a study that presented only one question: for example, “whether salinity gradients affect trait diversity”. Likewise, the traits from another study asking, for example, “whether trait differences increase leaf decomposition” were categorized as “effect traits.” When the study asked explicitly “whether salinity gradients affect trait diversity which, in turn, might affect ecosystem processes,” we considered those traits as response and effect traits, which were categorized as “both.” In cases in which authors did not explicitly inform trait type or asked questions about trait response or effect, we categorized the traits of that study as “undefined.”

We counted the number of studies using only response traits, but that mentioned how their findings related to ecosystem properties or functioning without further theoretical or empirical support. We considered such results to be incorrect since a response trait, by definition, indicates a response to environmental variation, which does not always imply an influence on ecosystem functioning (Raffard et al., 2017). The results were considered acceptable when the authors demonstrated a covariation between response and effect traits (Raffard et al., 2017) or between response traits and ecosystem functioning (Hordley et al., 2021). Moreover, we extracted the information showing whether a data set used (i) interspecific differences, which means the study used an average (e.g., mean body size), the maximum known value (e.g., maximum body size) or a categorical description (e.g., foraging guild) of a given species and if (ii) it included intraspecific trait variability within different individuals from the same species. Importantly, even those studies that measured several individuals from the same species but used on interspecific comparison without including within‐species variation in the analysis were considered “interspecific.” Therefore, to be included in the category “intraspecific,” the study should have explicitly used within‐species trait variability to understand trait response to or effect on the environment.

We used the periodic tables of niches (sensu Pianka, 1974, revisited by Winemiller et al., 2015) to categorize traits used by each study in one of the following niche dimensions: trophic, life history, habitat, defense, and metabolic. These niche dimensions are directly connected with ecological strategies affecting species performance and fitness. Winemiller et al. (2015) envisioned that Pianka's (1974) “periodic table of niches” may aid in identifying recurring patterns of convergent evolution and trait combinations in which cluster of species shares/prefers a given environmental state or performs a specified function (see also Appendix S2). Winemiller et al. (2015) argued that animal species traits may be arranged in few representative dimensions (as has already been observed for plants: Díaz et al., 2016), and, therefore, proposed five: habitat, life history, trophic, defense, and metabolic (see details about each dimension on the Table S3). At this stage, we first respected the category indicated by authors, for instance, trophic or life history dimension. Then, in cases in which authors did not indicate a specific niche dimension, we selected a dimension following Winemiller et al. (2015). In addition, when authors used morphological traits without explicitly linking them to one of the five dimensions, those traits were categorized as “undetermined morphological traits.” Lastly, traits without a clear indication in these dimensions were categorized as “other” (Table S3).

## 3 TRENDS, GAPS, AND BIASES IN THE STUDY OF ANIMAL FUNCTIONAL TRAITS

We extracted data from 1655 manuscripts published between 1999 and 2020 (Appendix S1: Figure S2). The number of published manuscripts that met our search criteria rose from an average of 6.25 per year between 1999 and 2011 to an average of 158 per year between 2011 and 2020. The first study using the keyword “functional diversity” or “functional trait” with animals was published by Olenin and Leppäkoski (1999). This manuscript used the term functional diversity to compare niche occupancy by non‐native benthic species in inlets and lagoons of the Baltic Sea. The most common author keywords were functional diversity (21.3% of all manuscripts), functional traits (17.9%), functional (7.9%), biodiversity (7.1%), diversity (5.7%), traits (5.0%), and ecosystem functioning (4.5%). Additional scientometric information about the extracted papers is available in the Appendix S1.

### 3.1 Taxonomic unit, resolution, and groups

We recovered 1790 data sets from 1655 studies that fit our eligibility criteria. Most data sets (84.4%) used traits at the species level (fine resolution), followed by genus (6.8%), and multiple taxonomic units (5.3%) (Appendix S1: Figure S3). In addition, the four most used taxonomic classifications were at the Class (31.1%), Order (24.1%), Phylum (17.1%), and Family (13.8%) levels (Appendix S1: Figure S4).

**FIGURE 1:**
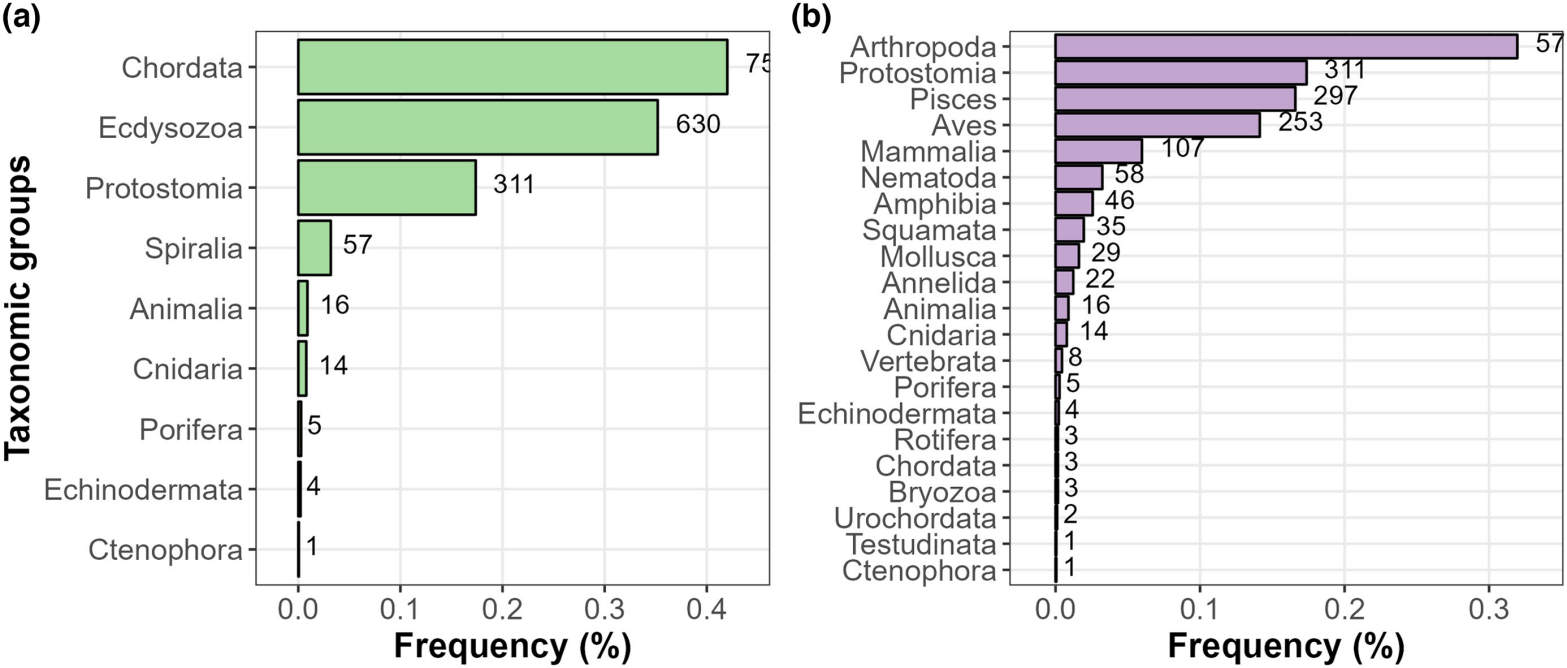
Number of data sets included in this systematic review grouped in lower and higher‐level taxonomic groups. Note that Animalia represents studies that used several groups (generally vertebrates and invertebrates) that did not allow for categorization at the lower levels. Furthermore, different groups are not nested within each other, so a data set labeled “Animalia” or “Vertebrata” did not necessarily, for example, include Pisces in their research.

We found that Vertebrata (42.1%), Ecdysozoa (35.1%), and Protostomia (17.3%) represented almost 95% of all studied data sets (Figure 1a). The five most studied taxonomic groups under this high‐level classification were Arthropoda (31.9%), Protostomia (17.3%), Pisces (16.6%), Aves (14.2%), and Mammalia (5.9%) (Figure 1b).

### 3.2 Trait selection (from trait type to niche dimensions)

**FIGURE 2:**
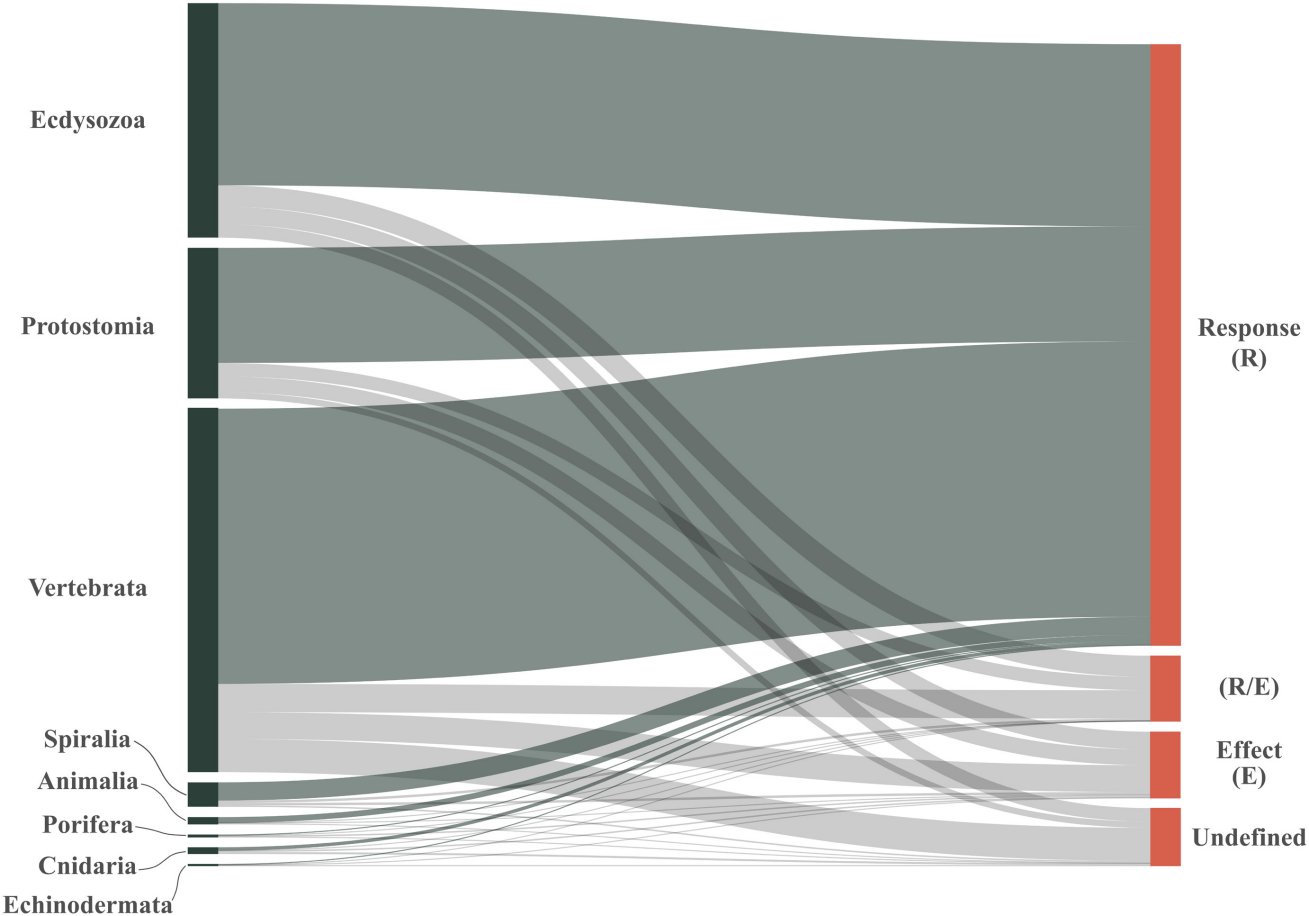
Alluvial diagram displaying the number of studies using different trait types (response, effect, response and effect, and undefined) in the following taxonomic groups: Ecdysozoa, Protostomia, Vertebrata, Spiralia, Animalia, Porifera, Cnidaria and Echinodermata.

We extracted different trait information from each data set, including: (i) trait type (response, effect, both, or undefined), (ii) whether intraspecific variability was included in the study, and (iii) which and how many niche dimensions were evaluated. We found that only 97 data sets (5.4%) examined intraspecific trait variation, while the remaining 1790 (94.6%) used trait averages (interspecific variation). Most data sets collected only response traits (79.4%), while 7.2% used effect traits, 7.3% adopted both response and effect traits, and 5.9% did not specify trait type. This low frequency of effect traits was similarly prevalent across taxonomic groups (Cnidaria, Ecdysozoa, Echinodermata, Protostomia, Spiralia, and Vertebrata), except for Porifera (Figure 2). When we combined trait type and whether or not a study included intraspecific variability with the geographical scale of the study, we discovered that (i) the effect traits were commonly used at both the local and global scales, (ii) undefined traits were mostly used at the global scale, and (iii) intraspecific variability was mostly used at the local scale (Appendix S1: Figure S5).

Furthermore, 235 (16.5%) of the 1422 studies that only used response traits addressed the potential impact on ecosystem functioning without providing justification for the link between the selected traits and their function. In fact, previous research has rarely examined effect traits in trait‐based animal ecology (e.g., arthropods: Brousseau et al., 2018; insects: Noriega et al., 2018). Therefore, relying on the assumption that a specific set of (usually response) traits significantly affects ecosystem functioning in most animals can lead to inconclusive results, as the relationship between the trait and its impact in the ecosystem is often unclear.

**FIGURE 3:**
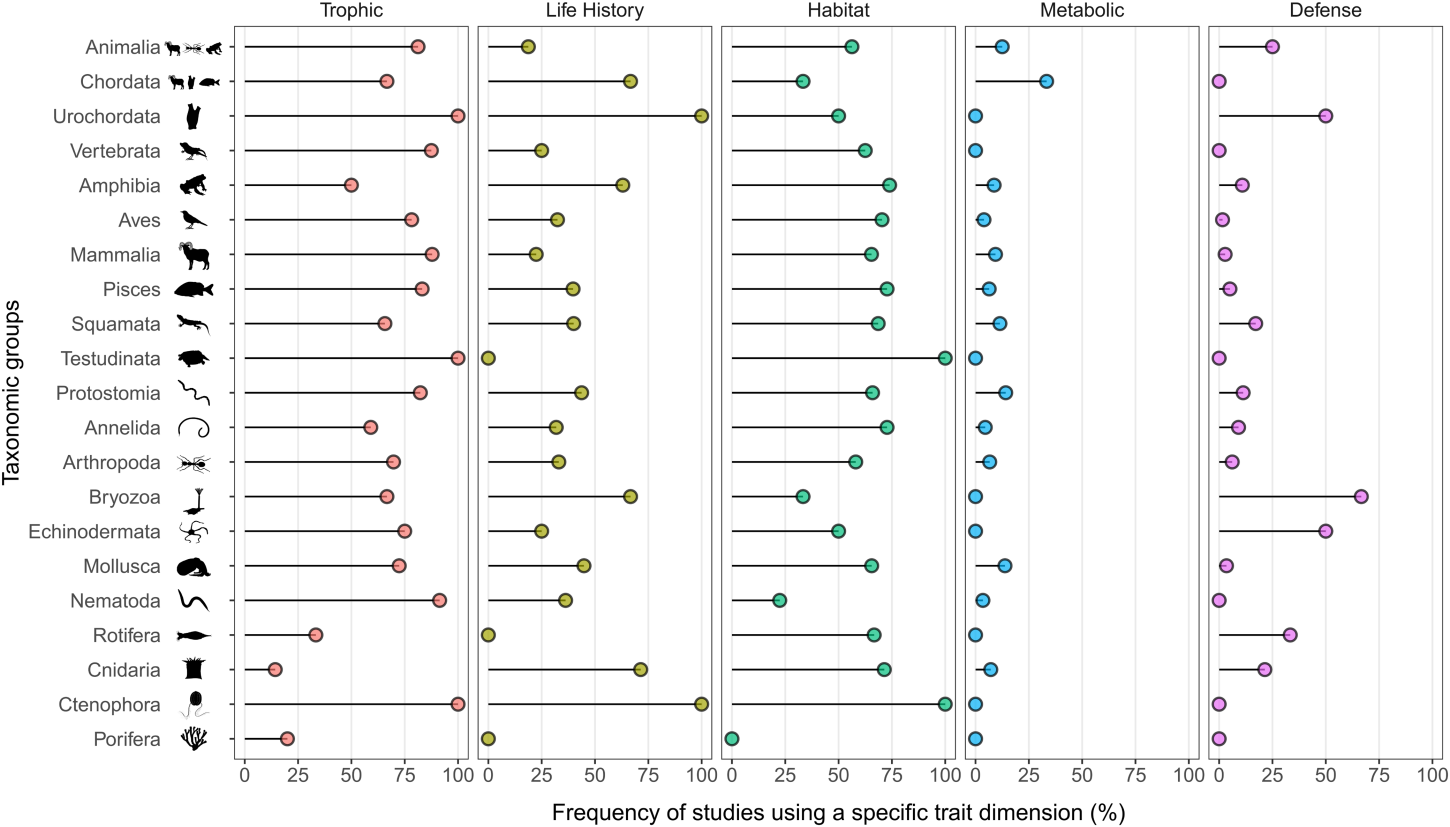
The number of studies in various taxonomic groups using different niche dimensions (trophic, life history, habitat, metabolic, and defense) aggregated in different taxonomic groups. This frequency ranges from 0 (no studies used that niche dimension) to 100% (all studies used that niche dimension for a given taxonomic group). Silhouettes are from http://phylopic.org/ and are licensed under Creative Commons license.

The most used niche dimensions were trophic (76.7% of the data sets), habitat (65.9%), life history (36.1%), metabolic (8.2%), and defense (6.9%). Although 468 data sets (28.3%) have used only one niche dimension, most of them used more than one niche dimension, varying from 2 (36.3%) to 3 (23.5%), 4 (4.7%), and 5 (0.7%). Importantly, even when analyzed with higher taxonomic levels, the trophic niche dimension was the most used (Figure 3). The only exception were studies with Porifera and Cnidaria that used mainly life history traits (Figure 3). Life history and habitat traits were also evenly used across different taxonomic groups, except for Porifera, Rotifera, and Testudinata (life history only). Conversely, data sets using metabolic traits (*n* = 136) were concentrated in 9 out of 18 taxonomic groups, being especially common in studies with Mollusca, Vertebrata, Cnidaria, Mammalia, and Nematoda (Figure 3). Lastly, defense traits were commonly used in studies with Bryozoa, Echinodermata, Rotifera, Cnidaria, and Squamata. However, this type of trait was not studied in 10 out of the 18 taxonomic groups (Figure 3).

Furthermore, 584 data sets (35.3%) used morphological traits without establishing a specific niche dimension, while 108 (6.5%) did not use any niche dimension at all. Even the most studied animals, such as vertebrates (20.7%), mammals (17.2%), birds (16%), and arthropods (15.4%) had a significant usage of unjustifiable morphological traits. Taken together, these results indicate many studies did not provide a specific niche dimension or ecological process underpinning trait selection. According to Winemiller et al. (2015), classifying traits into niche dimensions might be challenging since some traits may fall into many niche dimensions (e.g., body size). Even though categorizing traits into broad theoretical dimensions is challenging, it is imperative to show or discuss the functional basis underlying trait selection. For example, whereas Hall et al. (2019) used body size as a proxy for foraging range in bees, the same trait was used to represent life history strategies of fishes (Sternberg & Kennard, 2014). This is a critical limitation of animal research, as ignoring theory or the rationale underlying trait selection appears to be norm rather than exception (e.g., Hortal et al., 2015; Kearney et al., 2021; Winemiller et al., 2015), which emphasizes the challenge to advance trait selection in these organisms.

### 3.3 Ecosystem type and geographical extent

**FIGURE 4:**
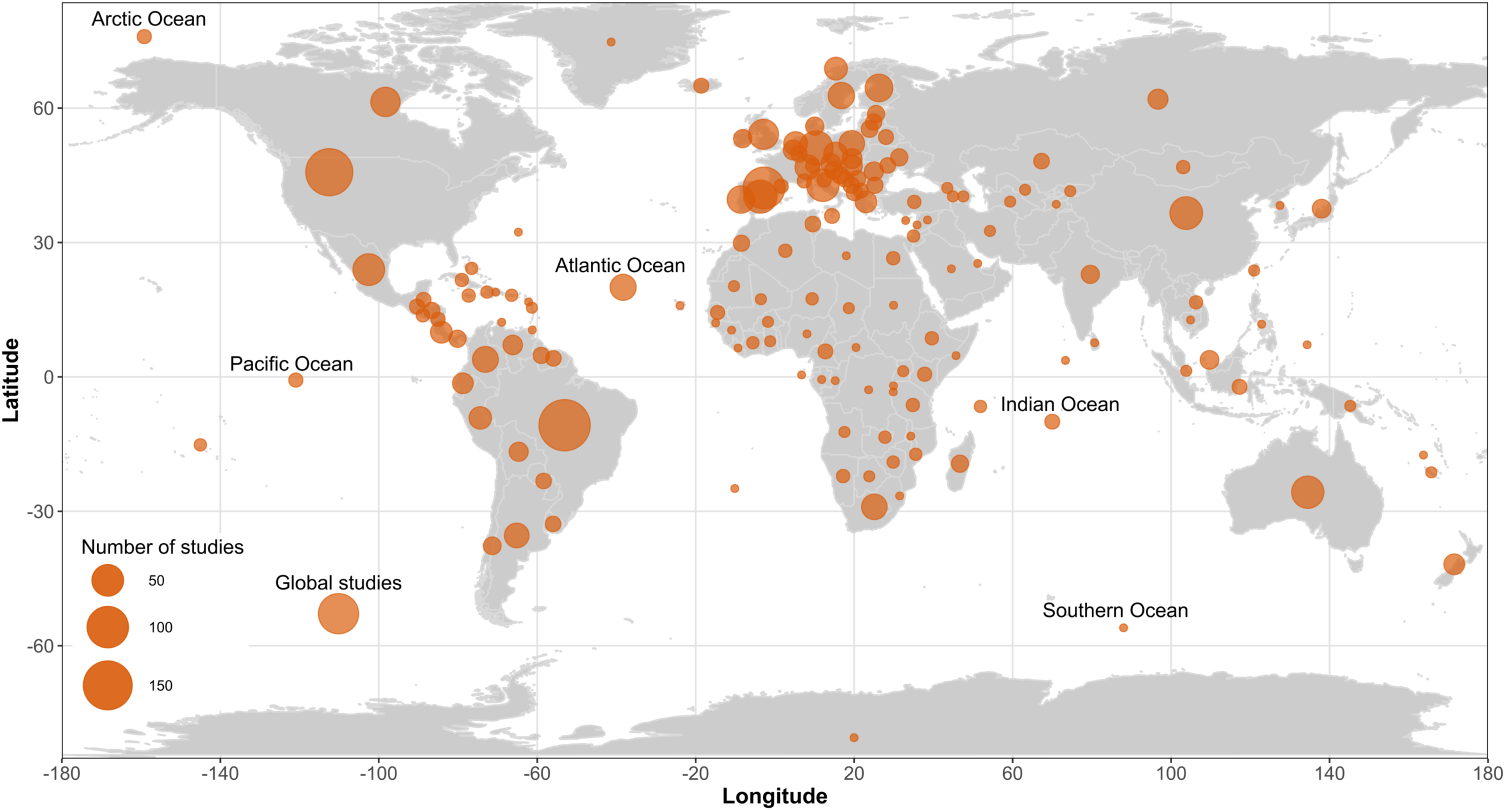
The terrestrial and marine regions of the 1540 studies, aggregated by country and ocean, are depicted on a map. Global studies have been placed at the bottom left of the map, as they differ depending on the examined country or continent.

We found 906 data sets (54.7%) studying terrestrial animals, followed by freshwater (27.7%) and marine (19%) organisms. These studies were performed in all continents and oceans, varying from local to global scales. For terrestrial and freshwater ecosystems, the most studied region was Europe (*n* = 503, 32.7%), followed by South America (19.1%), North America (15.8%), Asia (9%), Oceania (5.8%), and Africa (5.5%) (Figure 4). The Atlantic Ocean was the most studied marine biogeographical realm (2.7% of the studies), followed by the Mediterranean Sea (1.6%), Pacific Ocean (1.2%), Indian Ocean (0.6%), Arctic Ocean (0.4%), and the Red Sea (0.06%). We also mapped these studies in relation to zoogeographical regions and found that Palearctic (*n* = 642), Neotropical (*n* = 303), and Nearctic (*n* = 252) represented 66% of all data sets (Figure 4).

Our results reinforced the well‐known pattern of geographic biases in scientific efforts (Clarke et al., 2017; Culumber et al., 2019; Nuñez et al., 2021; Vasconcelos, 2023; Zuk, 2016). Previous studies on functional traits with specific taxa (Luiza‐Andrade et al., 2017) or themes (Hevia et al., 2017) already reported the predominance of studies conducted in Europe and North America. However, it is worth noting the relatively high number of studies in South America, likely due to the high scientific productivity of Brazil. It may be related to the fact that Brazil harbors some of the highest biodiversity in the world, has developed research centres, and expanded graduate programs across the country in the last decades (Almeida et al., 2021). Despite these advances, the scarcity of knowledge in tropical areas is critical, especially in the tropical regions of Africa and Southeast Asia, which jointly with the tropical region of South America, harbor most of the biodiversity of the planet (Loiseau et al., 2020). Trait‐based studies may provide useful subsides for ecological management and conservation policies (Malaterre et al., 2019). Thus, reducing geographic biases in research efforts on this subject is extremely important, especially in the current scenario in which mitigating the effect of environmental changes and the species extinctions is an urgent need.

## 4 THE TWO NEGLECTED ASPECTS OF TRAIT‐BASED ANIMAL ECOLOGY

Our study uncovered several biases in animal research using functional traits. We explicitly demonstrated that most research on animal functional traits focuses on response traits and uses trait averages, maximum values, or categorical traits to describe species attributes. Consequently, these studies are disregarding the significance of intraspecific variability on community dynamics and the direct effects of effect traits on ecosystem functioning. In fact, for most animals, the connection between traits and functions is rarely demonstrated.

The dominant strategy of studies at the community scale assumes that trait variation within and between populations (e.g., phenotypic plasticity, microgeographic adaptation) is unimportant because interspecific trait variation is more significant than intraspecific (Albert et al., 2011; Cianciaruso et al., 2009; Siefert et al., 2015). However, Bolnick et al. (2011) and Violle et al. (2012) suggested 10 years ago that incorporating trait variance is important to understand community dynamics and the implications for ecosystem functioning. The assumption that interspecific variation is usually larger than intraspecific trait variation is often simply not the case (Albert et al., 2011; Siefert et al., 2015). Albert et al. (2011) summarized why continuing to hold this assumption may be problematic: (i) the intraspecific variability may be larger than interspecific for a given trait (e.g., Siefert et al., 2015) and (ii) even when interspecific trait variation is greater than intraspecific, the variation within species may have an impact on community dynamics and ecosystem processes (e.g., Jung et al., 2010; Oliveira et al., 2021; Warzecha et al., 2016). For example, Jacob and Legrand (2021) demonstrated that, depending on the environmental gradient, the relative importance of trait variability within species changes from equal to outweighing the variation among ciliate species. In ants, thermal tolerance can vary among seasons within a species more than between species (Bujan et al., 2020) and among populations of the same species along elevational gradients as much as among species (Chick et al., 2020). Likewise, Siefert et al. (2015) demonstrated that intraspecific trait variability explained 32% of the total variation among plant communities. Even though many authors have repeatedly recommended the inclusion of intraspecific variability in trait‐based studies (Albert et al., 2011; Cam et al., 2002; Cianciaruso et al., 2009; Moretti et al., 2017; Raffard et al., 2017; Siefert et al., 2015), approximately 96% of animal research ignores this variation. This remarkable bias toward average traits implies that trait‐based animal ecology 2.0 should collect/investigate traits in various individuals of a given species within and between populations and communities along environmental gradients. The review by Green et al. (2022) also showed that trait‐based studies barely consider intraspecific variability and, what may be more concerning, none of the studies investigating global change issues used this information.

The relative contribution of intraspecific variability to trait diversity decreases with spatial extent (Chalmandrier et al., 2017; Siefert et al., 2015) and saturates at larger scales (“spatial variance partitioning hypothesis” sensu Albert et al., 2011). Therefore, whereas large scale research might be less influenced by concentrating simply on trait averages (Ibarra‐Isassi et al., 2023), neglecting intraspecific variability may have a considerable impact on the quality of studies conducted at regional and local scales. However, it is important to note that most empirical evidence comes from plants, highlighting the need for new animal research comparing at which spatial grain and extent intraspecific variability are relevant and how it varies with taxon groupings. Furthermore, most studies make use of databases that do not provide intraspecific information. Research on animals may benefit from the promising framework proposed by Carmona et al. (2016) for examining how intra‐ and interspecific trait variability differs among spatial scales. This framework built on the Hutchinsonian multidimensional niche concept uses trait probability density to quantify the contribution of different components of functional diversity (e.g., trait variance within and between populations, communities, or regions) across multiple spatial scales (Carmona et al., 2016).

Another aspect of trait‐based research on animals that is frequently overlooked is the use of effect traits (e.g., Brousseau et al., 2018 for arthropods). The issue stems from the fact that most studies of effect traits combine them with outputs of ecosystem properties. Gianuca et al. (2016), for example, found that body size diversity predicts zooplankton grazing efficiency and, hence, top‐down control. However, the great majority of research that uses response traits do not consider ecosystem aspects. This is particularly worrisome because nearly one‐fifth of the studies based only on response traits conclude that trait diversity impacts ecosystem processes or services without measuring them. Likewise, Noriega et al. (2018) demonstrated that knowledge connecting insect traits and ecosystem services is scarce and biased toward a few well‐known species. Furthermore, Hevia et al. (2017) reported that a few traits act as a response to environmental changes, while simultaneously affecting ecosystem services, when they investigated the relationship between 75 functional traits and ecosystem services (see also Hordley et al., 2021). As a consequence, if the selection of traits is not explicitly and clearly linked to data that supports the correlation between response traits and ecosystem properties/services, studies that connect response traits as a potential source of “effect” may be considered speculative.

Before evaluating if a set of response traits may have an impact on ecosystem properties, three limitations must be addressed to prevent arbitrary selection. First, selecting several traits might generate spurious correlation among traits affecting the quality of functional diversity estimation (Lefcheck et al., 2015; Zhu et al., 2017), consequently associating this diversity with ecosystem properties may be an analytical artifact. This is especially true in research areas where most evidence comes from observational data, which weakens our ability to explain the mechanism underlying trait–function correlation (see also Green et al., 2022). Two approaches used in the trait‐based ecology may be an exception to this limitation: those studies (i) seeking to characterize trait diversity at various sites or regions, and (ii) collecting many traits to identify the correlation between interspecific trait integration and gradients (Delhaye et al., 2020). The last example might be a useful method for selecting a set of potential traits that respond to the environment in unstudied organisms.

Second, selecting traits without considering the scale at which they are responsive may result in inaccurate interpretations (Green et al., 2022; Mlambo, 2014; Perronne et al., 2017; Rosado et al., 2016). The same trait, for example, may influence species responses to temperature over a latitudinal gradient (Grinnelian niche), but does not vary locally along a salinity gradient (Eltonian niche). The scale mismatches pointed out by Rosado et al. (2016) raises three critical questions that must be addressed before using functional traits from global data sets: What evidence exists to demonstrate that a particular trait is responsive to environmental variation across scales, from local to global? Additionally, what evidence is there to show that a specific trait affects ecosystem properties? Furthermore, if a correlation is evident, is there any evidence to suggest that it is dependent on the scale being considered? The negative implication of this bias is that the functional trait literature has not efficiently collected the essential knowledge to aid applied research in areas such as biodiversity management, global change ecology, and ecosystem services (Green et al., 2022; Hevia et al., 2017).

The third relevant limitation is that selecting both response and effect traits does not guarantee a correlation between them (Suding et al., 2008). This concern emphasizes the importance of animal research to mechanistically explore how the covariation between response and effect traits alter ecosystem properties (Raffard et al., 2017). A trait‐based response‐and‐effect framework holds promise for depicting those traits that respond to environmental changes and, as a result, influence the ecosystem (Roquer‐Beni et al., 2021; Suding et al., 2008). Furthermore, several effect traits may simultaneously affect a given ecosystem property (de Bello et al., 2010). As a result, when exploring traits as drivers of ecosystem processes, only traits with prior evidence linking trait variation to ecosystem functioning should be chosen.

## 5 IMPROVING TRAIT‐BASED ANIMAL ECOLOGY THROUGH EFFECTIVE TRAIT SELECTION

The trends and gaps that we have uncovered can assist in making informed decisions when choosing traits for animal‐based trait studies. Functional traits are commonly selected without proper reasoning, and are often influenced by biases such as relying on traits used in previous studies without justification or prioritizing traits that are readily available. Our logical rationale offers important considerations for creating best practices to standardize trait collection.

### 5.1 Which traits?

The first step is to clearly identify the meaning of traits in the study (if it is response or effect), derived from the study question (Malaterre et al., 2019). After deciding between response and effect traits (or both), the next step is confirming that there is evidence about trait–environment, trait–ecosystem, or environment–trait–ecosystem correlation—in essence, do the traits really affect the performance in nature? In fact, several studies used traits without an appropriate explanation about the underlying mechanism explaining how environmental change affects trait variation or how trait variation ultimately alters ecosystem functioning. Because selecting traits in studies with animals based on theory has rarely been the case (Kearney et al., 2021), defining whether your specific question requires a response or effect trait (or both) is a relevant step to avoid unconscious and inadequate trait selection (see Keller et al., 2023). Importantly, this question‐driven selection may result in a list of core or relevant traits needed to establish taxonomic group protocols (Brousseau et al., 2018). A shorter but relevant list of traits has at least two advantages: (i) it helps to avoid incorporating unknown traits that add no information or obscure the observed pattern/process and (ii) it limits the possibility of using correlated traits, which can impact some analytical methods (Lefcheck et al., 2015). It is crucial to highlight, however, that the debate over using a few or several traits remains open since recent research shows that using a single trait to explain variance in ecosystem functioning may perform better (Butterfield & Suding, 2013) or worse (Pakeman, 2014; van der Plas et al., 2020) than using multiple traits (see also Hortal et al., 2015).

In cases in which there is no previous evidence (experimental or observational), we suggest two directions: (i) develop a new experimental study to create new standards for a given taxonomic group or (ii) select only a subset of relevant traits with minimum evidence of trait–environment correlation (response traits). Furthermore, when there is no empirical support, it is desirable to prevent generalizing the impacts of trait or trait diversity on ecosystem functioning.

### 5.2 Validation of the spatial scale on which a trait responds to or impacts the environment

After selecting relevant traits and validating which trait type is used, it is essential to ask at which spatial scale a given trait is ecologically relevant? For example, what evidence is that the selected traits are connected to environmental variables operating at a given scale or that those traits influence ecosystem properties? This is particularly relevant when collecting trait information from databases that use a set of traits that respond to large‐scale environmental processes, but do not necessarily affect demographic rates locally (Perronne et al., 2017; Rosado et al., 2016). As with the preceding limitation, validating which process (and at what spatial scale) impacts the variation in the select trait(s), particularly in the Eltonian niche, is a critical next step in animal ecology (Cordlandwehr et al., 2013; Dehling & Stouffer, 2018; Rosado et al., 2016; Winemiller et al., 2015).

### 5.3 Trait averages and the return of the variance (again)

The broad statement that “trait variation among species is greater than trait variation within species” should be viewed with caution. Rather than considering this as a universal norm, future studies should account for intraspecific variability to estimate the true contribution of this variation to trait diversity. Furthermore, measuring the magnitude of intraspecific trait variation across different traits must help future studies decide whether including within‐species variance is necessary (Albert et al., 2011).

### 5.4 Periodic table of niches

**FIGURE 5:**
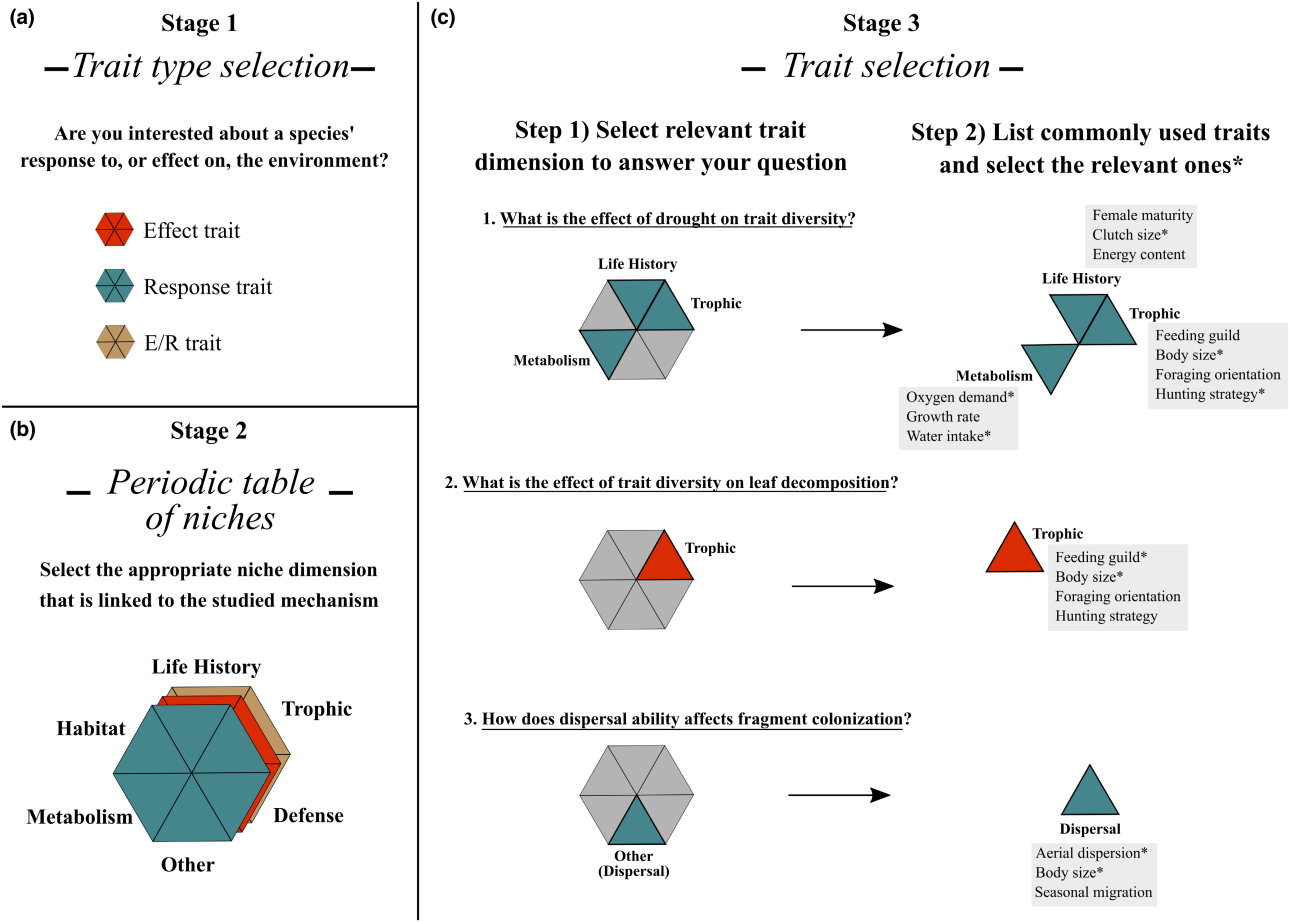
Trait collation guide: (a) traits can be organized into habitat, life history, trophic, defense, metabolism, and “other” dimension (e.g., dispersal) using the periodic table of niches. Furthermore, the type of trait chosen for the study (effect, response, or effect and response) is important. (b) The next relevant step is to choose the relevant trait type and dimension that correspond to your question; there is no necessity to select every niche dimension used in data sets or handbooks. (c) Lastly, make a list of all commonly used traits (available in handbooks, trait databases, reviews, etc.) and choose only those ones (in the figure hypothetically assigned with an asterisks) that have prior empirical evidence linking the relationship between environment–trait, trait–ecosystem, or environment–trait–ecosystem.

A step forward involves identifying the ecological processes and underlying niche dimensions associated with the research question before selecting traits to be included in the study. The periodic table of niches proposed by Winemiller et al. (2015) represents a synthetic way to classify traits into broad and discrete niche dimensions. Thinking about broad niche dimensions before the selection of the traits may help address the ecologically relevant features of organisms associated with the processes of interest. By establishing the most informative niche dimensions associated with the study question before choosing the specific candidate traits, researchers avoid falling into the trap of merely replicating previously used dimensions and traits without a rational basis. It is worrisome that several studies (35.3% of the data sets) did not suggest (even implicitly) the dimensions associated with the morphological traits used, leaving the interpretation of the functional role of the traits to the readers. Lastly, because the broad niche dimensions reflect the major challenges animals must deal with (Winemiller et al., 2015), their use as guide in trait selection represent a unified conceptual framework into which the myriad of specific traits that are used for different taxa can be accommodated. Figure 5 depicts a hypothesized workflow for defining trait type (Figure 5a), niche dimensions (Figure 5b), and selecting the relevant traits (Figure 5c) within the (study‐specific) informative dimensions. By adopting this approach and explicitly presenting niche dimensions and the traits chosen to describe them, the studies with different organisms and particular sets of traits become more integrated.

### 5.5 Effective trait selection

Once the study's question, trait type and trait validation, and the niche dimensions underlying the processes investigated are clearly specified, the following step is to identify the relevant traits that will be obtained/measured. Relevant traits are those associated with the underlying processes of the studied question. This is one the key steps in the design of trait‐based ecology studies—ask why these traits are relevant to organismal performance (or effect) and the ecological questions. However, whereas information on plant traits is standardized and broadly available (Kattge et al., 2020), the extent of the Raunkiæran shortfall remains a significant constraint for animals. Some well‐studied groups with high concordance in trait use such as mammals (Etard et al., 2020) may suggest a greater consensus among researchers in the set of traits to be used. Less studied groups (e.g., Echinodermata, Porifera, Cnidaria) or groups with low concordance in trait use may suggest the existence of an open field for new insights into trait selection processes as well as the need for functional morphology studies that addresses how organismal performance changes along environmental gradients. In both circumstances, high and low consensus does not ensure that studies with a specific taxonomic group are selecting traits based on past evidence concerning individual performance and fitness along environmental gradients (Laughlin & Laughlin, 2013). An unexplored approach in animal research, which is relatively well established in studies with plants (Díaz et al., 2016), might connect form and function impacting growth, survival, and reproduction underlying species ecological strategies (see, e.g., Arnold, 1983; Gibb et al., 2023; Junker et al., 2023).

It is crucial that there is consistency between the study question, the choice of traits, and the interpretation of patterns. However, using the “epistemic roadmap” outlined by Malaterre et al. (2019) can assist in organizing the concepts, definitions, theories, and empirical evidence related to trait selection and measurement. Additionally, Keller et al. (2023) have provided a clear set of 10 guidelines to enhance both the pre‐ and post‐trait collection process and improve trait‐based ecology research.

## 6 CONCLUSIONS AND PROSPECTS

Ecology has strong biases toward specific taxonomic groups (plants, birds) and geographical regions (temperate), which goes beyond studies with functional traits (Clarke et al., 2017; Culumber et al., 2019; Vasconcelos, 2023; Zuk, 2016). For example, almost half of the studies use vertebrates as model organisms. Almost 90% of the studied organisms belong to vertebrates, arthropods and macroinvertebrates. Likewise, ~55% of studies used terrestrial ecosystems, and one‐third were developed in the Palearctic region. While trait databases are incredibly valuable, they may perpetuate taxonomic and geographical biases because most investigated organisms/sites are also more likely to be shared/used. Nonetheless, traits obtained from these databases may impact our ability to investigate drivers of trait variation at the local scale where trait resolution may have a stark effect on functional diversity metrics (Silva et al., 2019). As previously advocated, scientific organizations, financial agencies, and curators of centralized databases may prioritize obtaining data from underrepresented locations and taxonomic groups (Culumber et al., 2019; Etard et al., 2020).

Inspired by previous reviews that highlighted several relevant limitations in trait‐based ecology (Green et al., 2022; Hevia et al., 2017), we hope this review will “propagate the good practices” advocated by Keller et al. (2023). Even though the use of functional traits in ecological literature has increased dramatically in the last decade, there are fundamental limitations that must be addressed to advance trait‐based animal ecology. We argue that filling these gaps will allow this research field to become more predictive in the future. Indeed, excellent recent studies defending complementary perspectives (Green et al., 2022; Streit & Bellwood, 2022) are most likely to move trait‐based ecology forward. Importantly, we need to bring light into the Raunkiæran shortfall by standardizing and facilitating trait selection and validation. On the other hand, we highlighted critical limitations (e.g., intraspecific trait variation, effect traits, scale validation) that must be considered to allow this field to successfully answer how environmental changes affect animals and their ability to provide ecosystem services and goods.

## AUTHOR CONTRIBUTIONS

**Thiago Gonçalves‐Souza:** Conceptualization (lead); data curation (lead); formal analysis (lead); methodology (equal); visualization (equal); writing – original draft (lead); writing – review and editing (lead). **Leonardo S. Chaves:** Data curation (equal); methodology (lead); writing – review and editing (equal). **Gabriel Boldorini:** Data curation (equal); formal analysis (equal); methodology (equal); writing – review and editing (equal). **Natália Ferreira:** Data curation (equal); methodology (equal); writing – review and editing (equal). **Reginaldo Gusmão:** Data curation (equal); methodology (equal); resources (lead); visualization (lead); writing – review and editing (equal). **Phamela Bernardes Perônico:** Data curation (equal); methodology (equal); writing – review and editing (equal). **Nate J Sanders:** Funding acquisition (supporting); visualization (supporting); writing – review and editing (equal). **Fabrício Barreto Teresa:** Conceptualization (equal); data curation (equal); methodology (equal); writing – original draft (equal); writing – review and editing (equal).

## CONFLICT OF INTEREST STATEMENT

The authors have no conflict of interest to declare.

## Supporting information


Appendix S1–S2

Table S1

Table S2

Table S3

## Data Availability

Data openly available in a public repository that issues datasets with https://doi.org/10.5061/dryad.g79cnp5v9.
