# Supplementary material for: Bringing light onto the Raunkiæran shortfall: A comprehensive review of traits used in functional animal ecology
Source: Ecol Evol. 2023 Apr 19;13(4):e10016. doi: 10.1002/ece3.10016 (PMC10115901; doi:10.1002/ece3.10016)
Supplement: Supplementary file 1 — Appendix S1–S2 [file ECE3-13-e10016-s002.docx]

**SUPPORTING INFORMATION**

**Bringing light into the Raunkiæran shortfall: a comprehensive review of traits used in functional animal ecology**

**Table S1**. Full list of the 1,790 datasets extracted from the 1,655 manuscripts using functional traits with animals (*.xlsx file)

**Table S2**. Studied taxon and its classification into low-level and high-level taxonomic group (*.xlsx file)

**Table S3**. Trait state, synonymized trait and niche dimensions of all traits found in the 1,790 datasets (*.xlsx file)

**Table S4**. Top 30 papers (and publication year) representing the most cited within the 1,540 studies. These papers were ranked by local citations (LCS), which represent the number of citations of these 30 papers exclusively found in the extracted studies (e.g., the first paper was cited in 509 out of 1,540 studies). (below)

**Shiny app for data visualization and download (beta version).**

<https://reginaldogusmao.shinyapps.io/Dashboard_traits/#section-home>

**Appendix S1. Scientometric map of functional animal ecology**

**Figure S1**. PRISMA flow diagram identifying the stages of the systematic review, which include article identification, duplication removal, screening, exclusion, and lastly included papers.

**Figure S2**. Number of articles published throughout time (1999 – 2020) that use the keywords “functional diversity" OR "functional trait" combined with several animal taxonomic groups.

**Figure S3**. Number of studies stratified by the taxonomic unit in which traits were measured/obtained.

**Figure S4**. Number of studies stratified by the taxonomic span, the most inclusive taxonomic group.

**Figure S5**. Frequency of trait type (response, effect, both or undefined) and use of intra- and interspecific trait variability among studies investigating different geographical scales. Circle size was standardized to varies between 0 and 100%.

**Table S4**. Top 30 papers (and publication year) representing the most cited within the 1,540 studies. These papers were ranked by local citations (LCS), which represent the number of citations of these 30 papers exclusively found in the extracted studies (e.g., the first paper was cited in 509 out of 1,540 studies).

**Appendix S2. Definition and categorization of niche dimensions**

**Appendix S1. Scientometric map of functional animal ecology**

The most influential papers (i.e., those cited in the 1,540 manuscripts with extracted data) including conceptual and methodological studies were Laliberte & Legendre (2010) (n = 509 citations), Villéger et al. (2008) (n = 487), McGill et al. 2006 (n = 408), Petchey et al. 2006 (n = 430), and Pavoine and Bonsall (2011) (n = 98). Furthermore, influential taxon-specific papers studied birds (Petchey et al. 2007: n = 102; Luck et al. 2012: n = 69; Tscharntke et al. 2008: n = 69), and fishes (Stuart-Smith et al. 2013: n = 97; Micheli et al. 2005: n = 85; Mouillot et al. 2013: n = 84) (Table S4). Likewise, the influential authors studied fishes (D. Mouillot: n = 960, S. Villéger: n =677, N. Mason: n = 612) and birds (O. Petchey: n = 595, Gaston: n = 555), but also published conceptual / methodological studies (P. Legendre: n = 510, E. Laliberté: n = 509). The affiliation of the first author of the published manuscripts were dominated by USA (n = 225 manuscripts), Brazil (156), France (133), Germany (95), United Kingdom (84), Australia (65), Canada (60), Spain (56), China (54), and Italy (50), from a list of 60 countries.

The 10 journals that published most papers in animal functional ecology were PLOS One (67 manuscripts), Ecology and Evolution (54), Ecological Indicators (52), Oecologia (38), Freshwater Biology (37), Ecography (36), Journal of Animal Ecology (36), Science of the Total Environment (33), Global Ecology and Biogeography (32), and Hydrobiology (32), from a list of 297 journals.

**
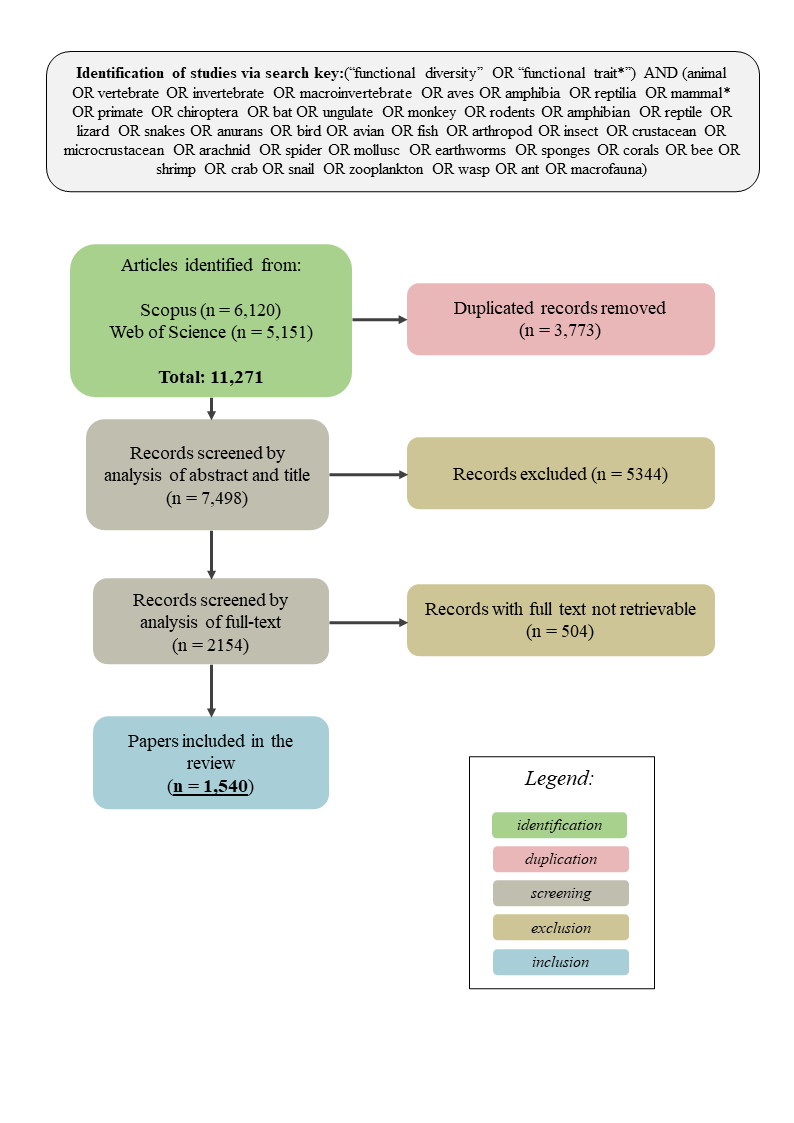
**

**Figure S1.**


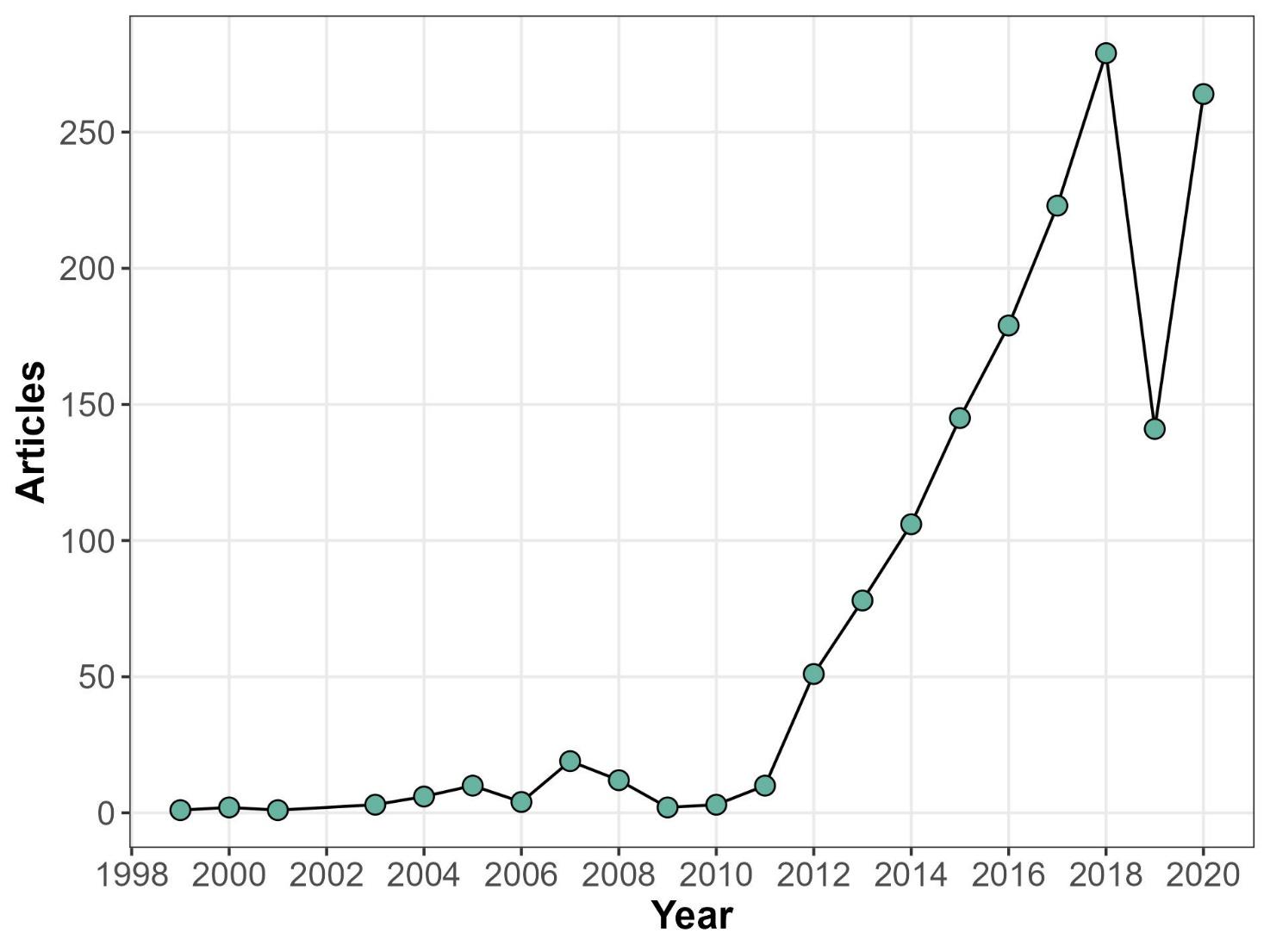


**Figure S2.**


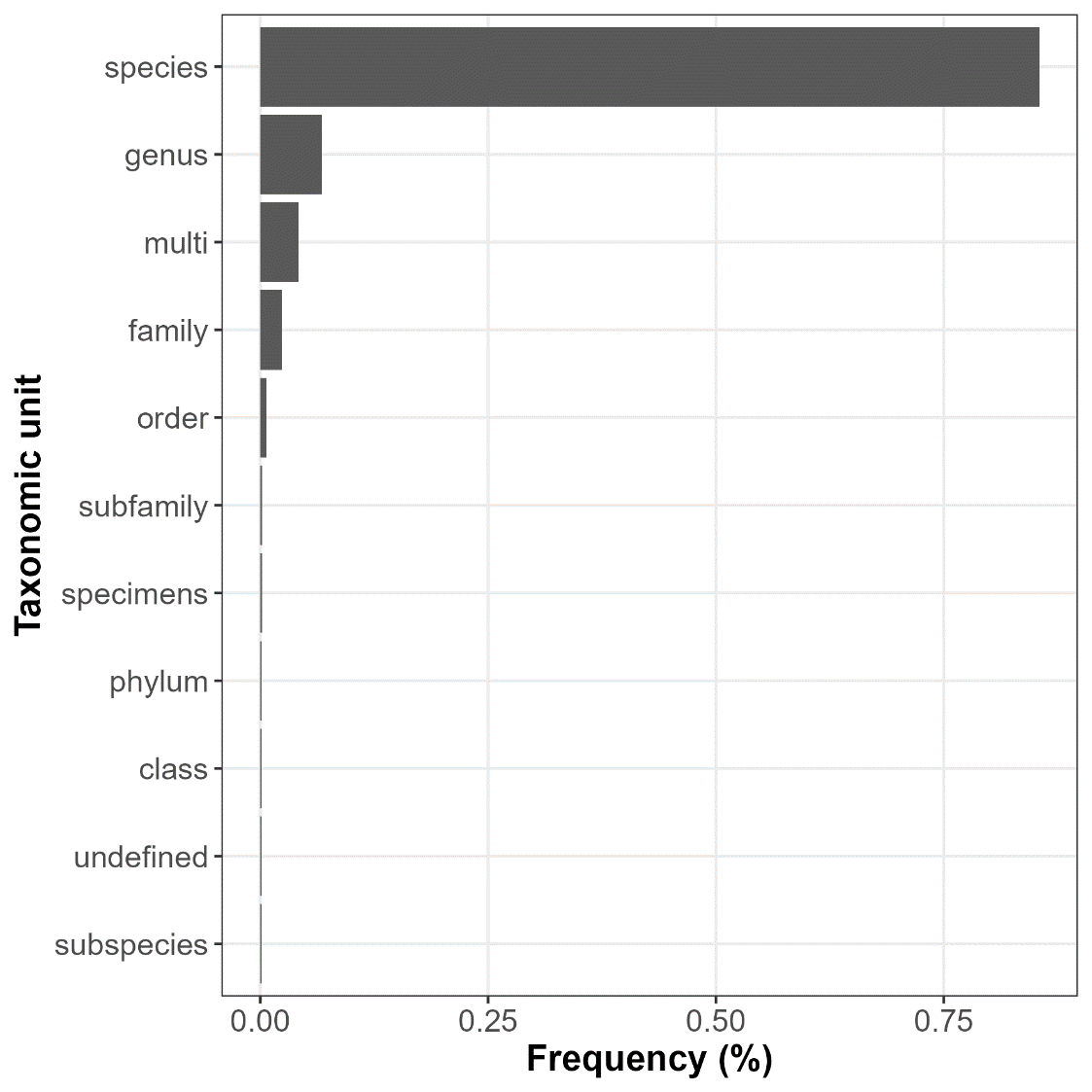


**Figure S3.**


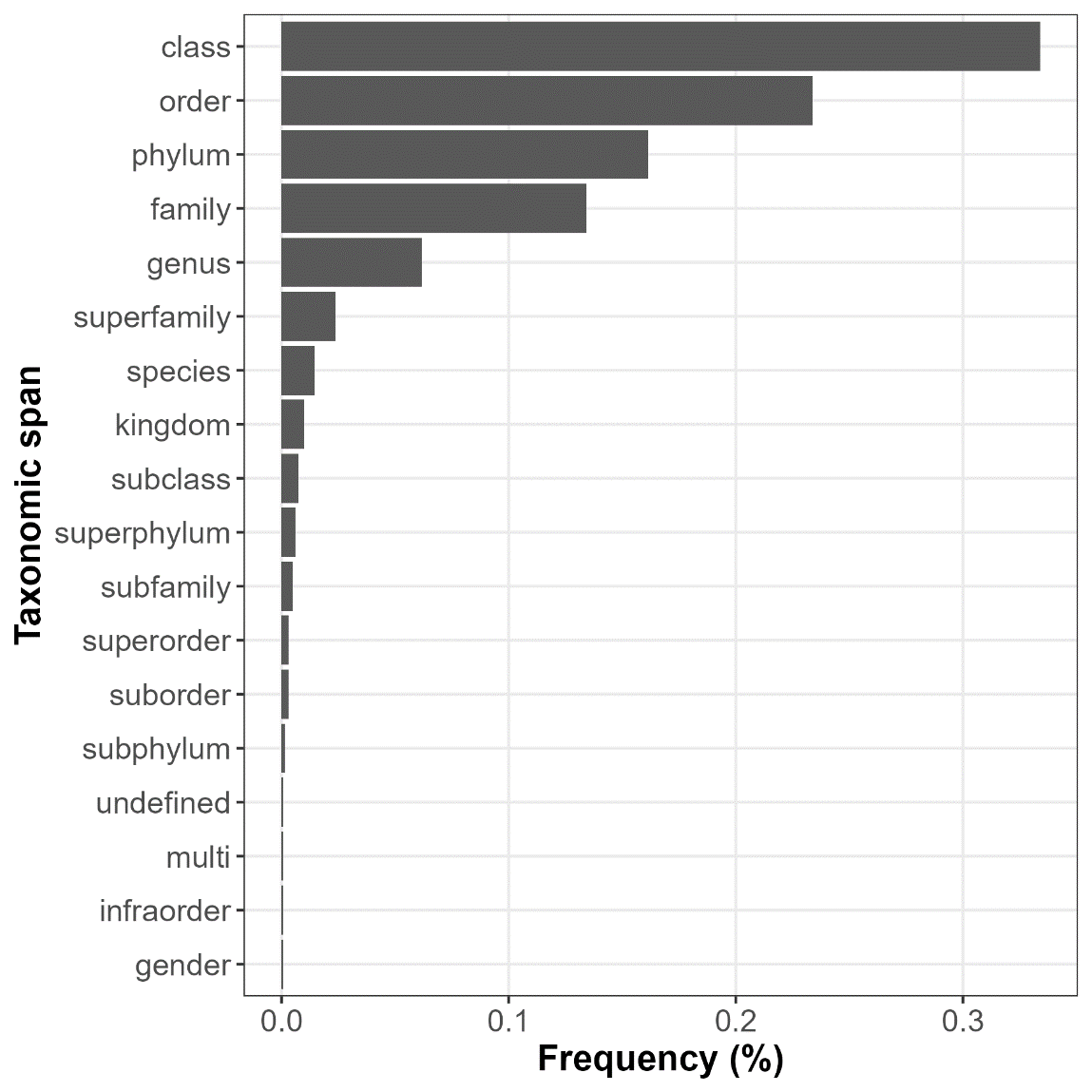


**Figure S4.**


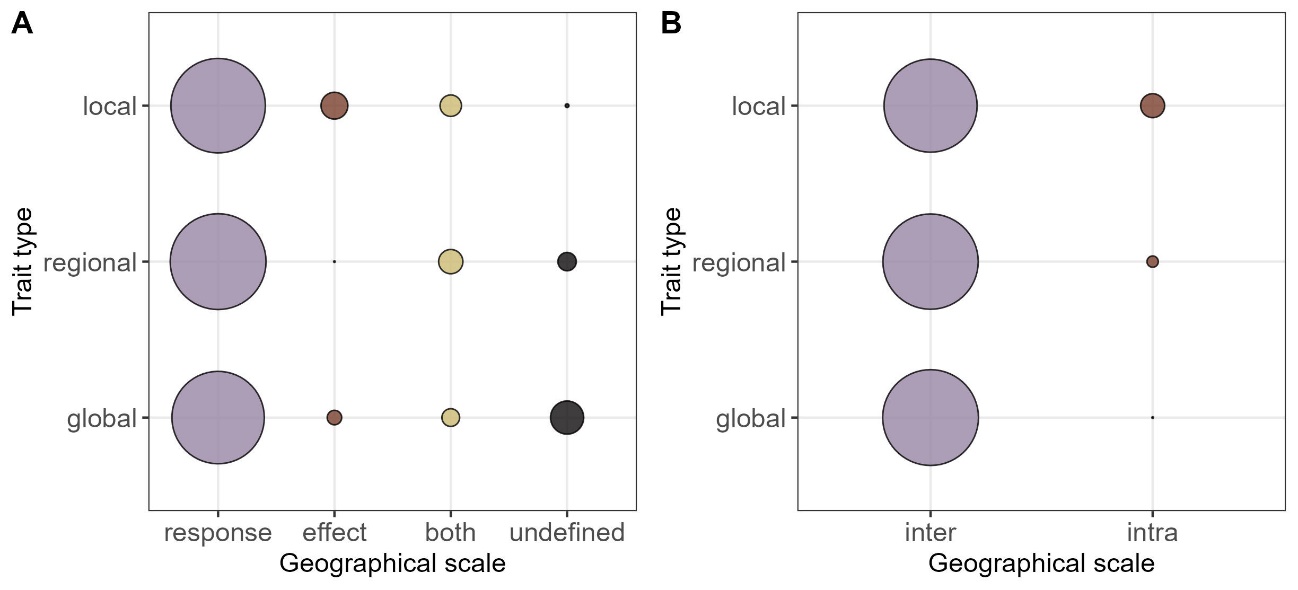


**Figure S5.**

**Table S4**. Top 30 papers (and publication year) representing the most cited within the 1,540 studies. These papers were ranked by local citations (LCS), which represent the number of citations of these 30 papers exclusively found in the extracted studies (e.g., the first paper was cited in 509 out of 1,540 studies).

| **Rank** | **Paper** | **Year** | **LCS** |
| --- | --- | --- | --- |
| 1 | LALIBERTE E, 2010, ECOLOGY | 2010 | 509 |
| 2 | VILLEGER S, 2008, ECOLOGY | 2008 | 487 |
| 3 | PETCHEY OL, 2006, ECOL LETT | 2006 | 430 |
| 4 | MCGILL BJ, 2006, TRENDS ECOL EVOL | 2006 | 408 |
| 5 | PETCHEY OL, 2007, J ANIM ECOL | 2007 | 102 |
| 6 | PAVOINE S, 2011, BIOL REV | 2011 | 98 |
| 7 | STUART-SMITH RD, 2013, NATURE | 2013 | 97 |
| 8 | MICHELI F, 2005, ECOL LETT | 2005 | 85 |
| 9 | MOUILLOT D, 2013, PLOS BIOL | 2013 | 84 |
| 10 | TSCHARNTKE T, 2012, BIOL REV | 2012 | 81 |
| 11 | LAVOREL S, 2008, FUNCT ECOL | 2008 | 73 |
| 12 | RICOTTA C, 2011, OECOLOGIA | 2011 | 72 |
| 13 | VIOLLE C, 2012, TRENDS ECOL EVOL | 2012 | 72 |
| 14 | TSCHARNTKE T, 2008, ECOLOGY | 2008 | 69 |
| 15 | LUCK GW, 2012, J ANIM ECOL | 2012 | 69 |
| 16 | BIHN JH, 2010, ECOLOGY | 2010 | 66 |
| 17 | VERBERK WCEP, 2013, FRESHW SCI | 2013 | 65 |
| 18 | NAEEM S, 2012, SCIENCE | 2012 | 62 |
| 19 | WINTER M, 2013, TRENDS ECOL EVOL | 2013 | 61 |
| 20 | STEVENS RD, 2003, ECOL LETT | 2003 | 58 |
| 21 | DIAZ S, 2013, ECOL EVOL | 2013 | 56 |
| 22 | LUCK GW, 2013, PLOS ONE | 2013 | 56 |
| 23 | CARDOSO P, 2011, PLOS ONE | 2011 | 53 |
| 24 | MOUILLOT D, 2011, PLOS ONE | 2011 | 53 |
| 25 | WINEMILLER KO, 2015, ECOL LETT | 2015 | 53 |
| 26 | MEYNARD CN, 2011, GLOBAL ECOL BIOGEOGR | 2011 | 51 |
| 27 | DE BELLO F, 2010, J VEG SCI | 2010 | 50 |
| 28 | GERISCH M, 2012, OIKOS | 2012 | 50 |
| 29 | MORETTI M, 2017, FUNCT ECOL | 2017 | 48 |
| 30 | LITCHMAN E, 2013, J PLANKTON RES | 2013 | 47 |

**Appendix S2. Definition and categorization of niche dimensions**

Winemiller et al. (2015) envisioned that Pianka’s (1974) “periodic table of niches” may aid in identifying recurring patterns of convergent evolution and trait combinations in which cluster of species shares/prefers a given environmental state or performs a specified function. Winemiller et al. (2015) argued that animal species traits may be arranged in few representative dimensions (as has already been observed for plants: Díaz et al., 2016), and therefore proposed five: habitat, life history, trophic, defence, and metabolic (see Table 1 in the page 742 of Winemiller et al., 2015). The ***habitat*** dimension determines whether an organism may survive in a suitable abiotic environment. This can be the temperature range that a species tolerates, as well as a spatial (e.g., depth), temporal (e.g., seasonal activity) and structural (e.g., substrate preference) abiotic condition. Winemiller et al. (2015) also suggested that the Grinnelian niche might be called the habitat dimension.

The ***life history*** dimension encapsulates the mechanisms that underpin the effects of environmental changes on species demography (Winemiller et al., 2015). Therefore, this dimension is related to, for example, energy and biomass allocation (e.g., C-S-R strategy) and reproductive time (e.g., generation time). The ***trophic*** dimension classifies organisms based on how they acquire and absorb resources for survival, development, and reproduction (Winemiller et al., 2015). This dimension covers feeding mechanisms (e.g., suction, raptorial), feeding behavior (e.g., hunting strategy), feeding/trophic guilds, and physiological strategies related to nutrition and energy storage (Winemiller et al., 2015). The ***defence*** (or ***survival***) dimension encompasses defensive strategies such as avoidance behaviour, weapons, poisons, and armour that reduce mortality caused by natural enemies such as predators and parasites, (Winemiller et al., 2015). The ***metabolic*** (or ***physiological***) dimension represents the allocations strategies of energy conservation, which includes thermoregulation, salinity tolerance, water conservation mechanisms, and others (Winemiller et al., 2015). It is essential to emphasize that some traits may be linked to more than one dimension. Body size, for example, can influence both a species’ the trophic position and the preferred habitat. As a result, in order to identify the appropriate niche dimensions, it is critical to understand the underlying mechanisms explaining trait-environment or trait-effect interactions.

**References**

Bihn, J. H., Gebauer, G., & Brandl, R. (2010). Loss of functional diversity of ant assemblages in secondary tropical forests. *Ecology*, *91*(3), 782–792. https://doi.org/10.1890/08-1276.1

Cardoso, P., Pekár, S., Jocqué, R., & Coddington, J. A. (2011). Global patterns of guild composition and functional diversity of spiders. *PLoS ONE*, *6*(6), e21710. https://doi.org/10.1371/journal.pone.0021710

De Bello, F., Lavergne, S., Meynard, C. N., Lepš, J., & Thuiller, W. (2010). The partitioning of diversity: Showing Theseus a way out of the labyrinth: Theseus and the partitioning of diversity. *Journal of Vegetation Science*, *21*(5), 992–1000. https://doi.org/10.1111/j.1654-1103.2010.01195.x

Díaz, S., Purvis, A., Cornelissen, J. H. C., Mace, G. M., Donoghue, M. J., Ewers, R. M., Jordano, P., & Pearse, W. D. (2013). Functional traits, the phylogeny of function, and ecosystem service vulnerability. *Ecology and Evolution*, *3*(9), 2958–2975. https://doi.org/10.1002/ece3.601

Gerisch, M., Agostinelli, V., Henle, K., & Dziock, F. (2012). More species, but all do the same: Contrasting effects of flood disturbance on ground beetle functional and species diversity. *Oikos*, *121*(4), 508–515. https://doi.org/10.1111/j.1600-0706.2011.19749.x

Laliberté, E., & Legendre, P. (2010). A distance-based framework for measuring functional diversity from multiple traits. *Ecology*, *91*(1), 299–305. https://doi.org/10.1890/08-2244.1

Lavorel, S., Grigulis, K., McIntyre, S., Williams, N. S. G., Garden, D., Dorrough, J., Berman, S., Quétier, F., Thébault, A., & Bonis, A. (2007). Assessing functional diversity in the field – methodology matters! *Functional Ecology*, *0*(0), 071124124908001-??? https://doi.org/10.1111/j.1365-2435.2007.01339.x

Litchman, E., Ohman, M. D., & Kiørboe, T. (2013). Trait-based approaches to zooplankton communities. *Journal of Plankton Research*, *35*(3), 473–484. https://doi.org/10.1093/plankt/fbt019

Luck, G. W., Carter, A., & Smallbone, L. (2013). Changes in bird functional diversity across multiple land uses: Interpretations of functional redundancy depend on functional group identity. *PLoS ONE*, *8*(5), e63671. https://doi.org/10.1371/journal.pone.0063671

Luck, G. W., Lavorel, S., McIntyre, S., & Lumb, K. (2012). Improving the application of vertebrate trait-based frameworks to the study of ecosystem services: Vertebrate trait-based frameworks. *Journal of Animal Ecology*, *81*(5), 1065–1076. https://doi.org/10.1111/j.1365-2656.2012.01974.x

Mcgill, B., Enquist, B., Weiher, E., & Westoby, M. (2006). Rebuilding community ecology from functional traits. *Trends in Ecology & Evolution*, *21*(4), 178–185. https://doi.org/10.1016/j.tree.2006.02.002

Meynard, C. N., Devictor, V., Mouillot, D., Thuiller, W., Jiguet, F., & Mouquet, N. (2011). Beyond taxonomic diversity patterns: How do α, β and γ components of bird functional and phylogenetic diversity respond to environmental gradients across France?: Multiple facets of diversity. *Global Ecology and Biogeography*, *20*(6), 893–903. https://doi.org/10.1111/j.1466-8238.2010.00647.x

Micheli, F., & Halpern, B. S. (2005). Low functional redundancy in coastal marine assemblages. *Ecology Letters*, *8*(4), 391–400. https://doi.org/10.1111/j.1461-0248.2005.00731.x

Moretti, M., Dias, A. T. C., Bello, F., Altermatt, F., Chown, S. L., Azcárate, F. M., Bell, J. R., Fournier, B., Hedde, M., Hortal, J., Ibanez, S., Öckinger, E., Sousa, J. P., Ellers, J., & Berg, M. P. (2017). Handbook of protocols for standardized measurement of terrestrial invertebrate functional traits. *Functional Ecology*, *31*(3), 558–567. https://doi.org/10.1111/1365-2435.12776

Mouillot, D., Bellwood, D. R., Baraloto, C., Chave, J., Galzin, R., Harmelin-Vivien, M., Kulbicki, M., Lavergne, S., Lavorel, S., Mouquet, N., Paine, C. E. T., Renaud, J., & Thuiller, W. (2013). Rare species support vulnerable functions in high-diversity ecosystems. *PLoS Biology*, *11*(5), e1001569. https://doi.org/10.1371/journal.pbio.1001569

Mouillot, D., Villéger, S., Scherer-Lorenzen, M., & Mason, N. W. H. (2011). Functional structure of biological communities predicts ecosystem multifunctionality. *PLoS ONE*, *6*(3), e17476. https://doi.org/10.1371/journal.pone.0017476

Naeem, S., Duffy, J. E., & Zavaleta, E. (2012). The functions of biological diversity in an age of extinction. *Science*, *336*(6087), 1401–1406. https://doi.org/10.1126/science.1215855

Pavoine, S., & Bonsall, M. B. (2011). Measuring biodiversity to explain community assembly: A unified approach. *Biological Reviews*, *86*(4), 792–812. https://doi.org/10.1111/j.1469-185X.2010.00171.x

Petchey, O. L., Evans, K. L., Fishburn, I. S., & Gaston, K. J. (2007). Low functional diversity and no redundancy in British avian assemblages. *Journal of Animal Ecology*, *76*(5), 977–985. https://doi.org/10.1111/j.1365-2656.2007.01271.x

Petchey, O. L., & Gaston, K. J. (2006). Functional diversity: Back to basics and looking forward. *Ecology Letters*, *9*(6), 741–758. <https://doi.org/10.1111/j.1461-0248.2006.00924.x>

Pianka, E.R. (1974). *Evolutionary Ecology*. First Ed., Harper and Row, New York, NY.

Ricotta, C., & Moretti, M. (2011). CWM and Rao’s quadratic diversity: A unified framework for functional ecology. *Oecologia*, *167*(1), 181–188. https://doi.org/10.1007/s00442-011-1965-5

Stevens, R. D., Cox, S. B., Strauss, R. E., & Willig, M. R. (2003). Patterns of functional diversity across an extensive environmental gradient: Vertebrate consumers, hidden treatments and latitudinal trends: Gradients of functional diversity. *Ecology Letters*, *6*(12), 1099–1108. https://doi.org/10.1046/j.1461-0248.2003.00541.x

Stuart-Smith, R. D., Bates, A. E., Lefcheck, J. S., Duffy, J. E., Baker, S. C., Thomson, R. J., Stuart-Smith, J. F., Hill, N. A., Kininmonth, S. J., Airoldi, L., Becerro, M. A., Campbell, S. J., Dawson, T. P., Navarrete, S. A., Soler, G. A., Strain, E. M. A., Willis, T. J., & Edgar, G. J. (2013). Integrating abundance and functional traits reveals new global hotspots of fish diversity. *Nature*, *501*(7468), 539–542. https://doi.org/10.1038/nature12529

Tscharntke, T., Sekercioglu, C. H., Dietsch, T. V., Sodhi, N. S., Hoehn, P., & Tylianakis, J. M. (2008). Landscape constraints on functional diversity of birds and insects in tropical agroecosystems. *Ecology*, *89*(4), 944–951. https://doi.org/10.1890/07-0455.1

Tscharntke, T., Tylianakis, J. M., Rand, T. A., Didham, R. K., Fahrig, L., Batáry, P., Bengtsson, J., Clough, Y., Crist, T. O., Dormann, C. F., Ewers, R. M., Fründ, J., Holt, R. D., Holzschuh, A., Klein, A. M., Kleijn, D., Kremen, C., Landis, D. A., Laurance, W., … Westphal, C. (2012). Landscape moderation of biodiversity patterns and processes—Eight hypotheses. *Biological Reviews*, *87*(3), 661–685. https://doi.org/10.1111/j.1469-185X.2011.00216.x

Verberk, W. C. E. P., van Noordwijk, C. G. E., & Hildrew, A. G. (2013). Delivering on a promise: Integrating species traits to transform descriptive community ecology into a predictive science. *Freshwater Science*, *32*(2), 531–547. https://doi.org/10.1899/12-092.1

Villéger, S., Mason, N. W. H., & Mouillot, D. (2008). New multidimensional functional diversity indices for a multifaceted framework in functional ecology. *Ecology*, *89*(8), 2290–2301. https://doi.org/10.1890/07-1206.1

Violle, C., Enquist, B. J., McGill, B. J., Jiang, L., Albert, C. H., Hulshof, C., Jung, V., & Messier, J. (2012). The return of the variance: Intraspecific variability in community ecology. *Trends in Ecology & Evolution*, *27*(4), 244–252. https://doi.org/10.1016/j.tree.2011.11.014

Winemiller, K. O., Fitzgerald, D. B., Bower, L. M., & Pianka, E. R. (2015). Functional traits, convergent evolution, and periodic tables of niches. *Ecology Letters*, *18*(8), 737–751. https://doi.org/10.1111/ele.12462

Winter, M., Devictor, V., & Schweiger, O. (2013). Phylogenetic diversity and nature conservation: Where are we? *Trends in Ecology & Evolution*, *28*(4), 199–204. https://doi.org/10.1016/j.tree.2012.10.015
